# Adrenomedullin levels in patients with Familial Mediterranean Fever: a long term follow-up

**DOI:** 10.1186/1546-0096-13-S1-P99

**Published:** 2015-09-28

**Authors:** A Polat, C Saglam, YG Kurt, G Basbozkurt, B Sozeri, I Dursun, O Kasapcupur, H Peru, D Simsek, Z Gunduz, E Unsal, F Gok, S Ozen, E Demirkaya

**Affiliations:** 1 FMF Arthritis Vasculitis and Orphan Disease Research in Paediatric Rheumatology (FAVOR), Turkey

## Introduction

Familial Mediterranean Fever (FMF) is the most common periodic fever syndrome, characterized by recurrent fever and serositis attacks. It has been shown that there might be an ongoing subclinical inflammation between attacks. Adrenomedullin (ADM) is synthesized in endothelium, and has been shown to have high levels in patients with inflammation such as FMF. Colchicine is the treatment of choice and given once or twice daily depending on expert opinion.

## Objectives

In this study, it was aimed to investigate ADM as a marker for inflammation in pediatric patients with FMF who are using colchicine in different dosage schema.

## Methods

Pediatric patients diagnosed with clinically and genetically confirmed FMF diagnosis were included in the study. The Colchicine was started in one or two doses randomly. The clinical and laboratory parameters were assessed on six clinical visits made every two months. After the third visit the dosing schema was changed to twice or once depending on the schema at the beginning.

## Results

A total of 37 patients were included in the study. Mean age of patients was 7.78±2.00 years, mean age at disease onset was 5.05 ± 3.04 years and mean age at diagnosis was 7.51 ± 2.66 years. Twenty patients received colchicine in once daily dosage while 17 patients had in twice daily dosage at the beginning of the study. There were 10 patients with heterozygote and 27 with homozygote MEFV mutations. After the treatment was started all patients demonstrated improvement in clinical and laboratory findings such as erythrocyte sedimentation rate and C-reactive protein. However, ADM levels did not show any correlation with ESR and CRP levels. Mean ADM levels in six consequtive visits were as follows, first 322.19±161.92 ng/L; second 330.50±189.63 ng/L; third 339.54±168.03 ng/L; forth 378.11±177.63 ng/L; fifth 328.91±172.30 ng/L and sixth 326.25±165.87 ng/L. ADM levels were similar in all visits (*p*=0.954) and did not show any difference between the first and second three visits i.e. before and after changing the dosage schema (*p*=0.593).

## Conclusion

The results indicated that patients using colchicine in once or twice daily doses did not demonstrate any difference considering clinical and laboratory findings and had similar effects in controlling disease manifestations. ADM levels did not demonstrate any alterations in all visits which may suggest the continuation of subclinical inflammation in these patients.

